# Evidence that HIV-1 restriction factor SAMHD1 facilitates differentiation of myeloid THP-1 cells

**DOI:** 10.1186/s12985-015-0425-y

**Published:** 2015-11-25

**Authors:** Loic Dragin, Soundasse Munir-Matloob, Jeanne Froehlich, Marina Morel, Adèle Sourisce, Hichem Lahouassa, Karine Bailly, Marianne Mangeney, Bertha Cecilia Ramirez, Florence Margottin-Goguet

**Affiliations:** Inserm, U1016, Institut Cochin, 22 rue Méchain, 75014 Paris, France; CNRS, UMR8104, Paris, France; Université Paris Descartes, Sorbonne Paris Cité, Paris, France

**Keywords:** HIV, SAMHD1, Restriction factor, Differentiation, Cell cycle, Myeloid cells, T cells

## Abstract

**Background:**

SAMHD1 counteracts HIV-1 or HIV-2/SIVsmm that lacks Vpx by depleting the intracellular pool of nucleotides in myeloid cells and CD4+ quiescent T cells, thereby inhibiting the synthesis of retroviral DNA by reverse transcriptase. Depletion of nucleotides has been shown to underline the establishment of quiescence in certain cellular systems. These observations led us to investigate whether SAMHD1 could control the transition between proliferation and quiescence using the THP-1 cell model.

**Findings:**

The entry of dividing THP-1 myeloid cells into a non-dividing differentiated state was monitored after addition of phorbol-12-myristate-13-acetate (PMA), an inducer of differentiation. Under PMA treatment, cells overexpressing SAMHD1 display stronger and faster adhesion to their support, compared to cells expressing a catalytically inactive form of SAMHD1, or cells depleted of SAMHD1, which appear less differentiated. After PMA removal, cells overexpressing SAMHD1 maintain low levels of cyclin A, in contrast to other cell lines. Interestingly, SAMHD1 overexpression slightly increases cell adhesion even in the absence of the differentiation inducer PMA. Finally, we found that levels of SAMHD1 are reduced in proliferating primary CD4+ T cells after T cell receptor activation, suggesting that SAMHD1 may also be involved in the transition from a quiescent state to a dividing state in primary T cells.

**Conclusions:**

Altogether, we provide evidence that SAMHD1 may facilitate some aspects of THP-1 cell differentiation. Restriction of HIV-1 by SAMHD1 may rely upon its ability to modify cell cycle parameters, in addition to the direct inhibition of reverse transcription.

**Electronic supplementary material:**

The online version of this article (doi:10.1186/s12985-015-0425-y) contains supplementary material, which is available to authorized users.

## Findings

Immune quiescent cells display reduced susceptibility to HIV-1 productive infection, due to several quiescence-related phenomena such as cytoskeleton organization or low viral transcription. The activity of several restriction factors also contributes to viral inhibition by blocking specific steps of the viral life cycle. Among them, sterile alpha motif and HD-domain containing protein 1 (SAMHD1) inhibits both HIV-1 and Vpx-deleted HIV-2/SIVsmm viruses at the level of reverse transcription [1, 2]. SAMHD1 reduces the intracellular pool of dNTP through its dNTP hydrolase activity in myeloid and quiescent CD4+ T cells [3–7]. Recent studies report that SAMHD1 also has an RNase activity targeting HIV-1 genomic RNA [8]. The dNTPase activity of the protein is dependent on an intact phosphodiesterase HD domain [1, 2]. Though HIV-1 is sensitive to SAMHD1, it has not developed a viral weapon to counteract this hindrance. In contrast, HIV-2/SIVsmm encodes for the Vpx auxiliary protein, which induces SAMHD1 degradation by hijacking the Cul4A-DDB1 ubiquitin ligase through DCAF1 binding [1, 2].

Several reports point to a role of SAMHD1 in tumorigenesis and in cell cycle progression. In particular, SAMHD1 has been found mutated in chronic lymphocytic leukemia (CLL) [9]. In CLL patient cells, SAMHD1 expression is often reduced as it is the case in several cancer cell lines [9]. Whole sequencing has also suggested that SAMHD1 could be mutated in malignancies [10]. Regarding cell cycle progression, proliferation in HeLa cells is favored by the dNTPase defective SAMHD1 HD/AA mutant [9]. In fibroblasts, SAMHD1 has been shown to be variously expressed along the cell cycle, maximally during quiescence and minimally during S-phase, which correlates with the need for dNTP during DNA replication [11]. Apart from SAMHD1, several reports suggest that suppression of nucleotide metabolism underlines the establishment of a quiescent state and promotes genomic instability [12–14]. Altogether, we brought up the hypothesis that SAMHD1 may facilitate the entry into or the maintenance of quiescence. To test our hypothesis, we used suspension cultures of the monocytic cell line THP-1 that differentiate into a macrophage-like phenotype under phorbol-12-myristate-13-acetate (PMA) treatment and therefore transit from a dividing state to a quiescent state. PMA-induced differentiation of THP-1 cells results, among other things, in cell morphological changes, adhesion to the plastic surface and expression of macrophage surface markers such as CD11a, CD11b and ICAM1 [15, 16]. In addition, THP-1 cells acquire the ability to restrict HIV infection along differentiation, following SAMHD1 dephosphorylation on a specific threonine [17–19].

We established THP-1 myeloid cell lines stably overexpressing HA-tagged wt SAMHD1 or the catalytic mutant form of the protein (HD/AA, referred to SAMHD1μ on the figures) or cells depleted of SAMHD1 (shSAMHD1). As previously reported, the promoter from the pLenti vector used for SAMHD1 expression is sensitive to PMA [4]. In the absence of PMA, exogenous SAMHD1 is poorly expressed, while in PMA-treated cells, levels of SAMHD1 and SAMHD1 HD/AA increased equally (Additional file 1: Figure S1A).

**Fig. 1:**
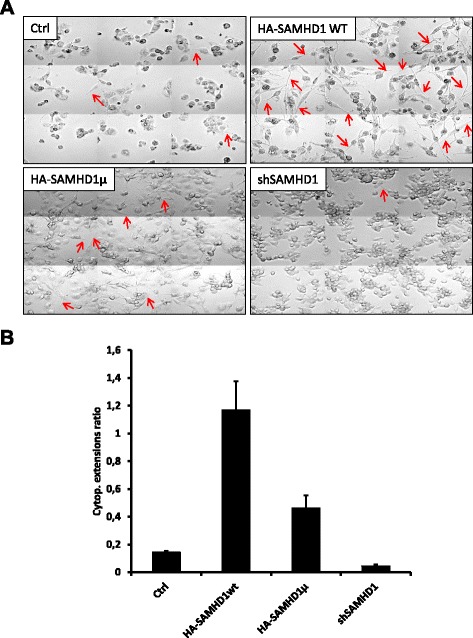
SAMHD1 overexpression promotes PMA-induced differentiation-related morphological phenotype of THP-1 cells. THP-1 control cells and monoclonal cell lines stably expressing HA-tagged SAMHD1 (wild-type or the HD/AA catalytically inactive mutant) or shRNA targeting SAMHD1 were differentiated by addition of 65 nM of PMA in the culture medium (2.10^5 cells/ml). 24 h after PMA removal, cells were left in PMA-free medium for several days. (**a**) Cell morphology was observed with a Zeiss 5 microscope (Gx20). The pictures shown here were taken 72 h after PMA addition. The results are representative of three independent experiments. (**b**) For each cell line, cytoplasmic extensions of cells 72 h after PMA addition were counted and normalized with regards to cell numbers (approximately 300 cells in each condition). The cytoplasmic extensions ratios obtained result from two independent experiments

PMA-mediated THP-1 differentiation is visible under the microscope with the appearance of multiple morphological changes, including the transition from a round shape to a fibroblast-like shape, the appearance of membrane extensions and cell adhesion to the support (Additional file 2: Figure S2). Following PMA treatment, cells stably expressing HA-tagged SAMHD1 displayed a fibroblast-like shape and multiple extensions faster than control cells (Ctrl) or cells depleted of SAMHD1 (shSAMHD1) that remained in a round shape (Fig. 1a and Additional file 2: Figure S2 for the kinetics of these changes). Cells stably expressing the HD/AA mutant had an intermediate phenotype. For each cell line we counted the cytoplasmic extensions appearing over time after PMA-induced differentiation. Determination of the ratios between the number of extensions and the number of cells in each condition showed an increase of differentiation-associated cytoplasmic extensions in cells overexpressing HA-SAMHD1 compared to control cells (Fig. 1b). As suspected, cells overexpressing the HD/AA catalytic mutant showed an intermediary phenotype, with more extensions than control cells but less than cells overexpressing the wild-type protein. As a corollary, SAMHD1 depleted cells displayed less of these extensions.

**Fig. 2:**
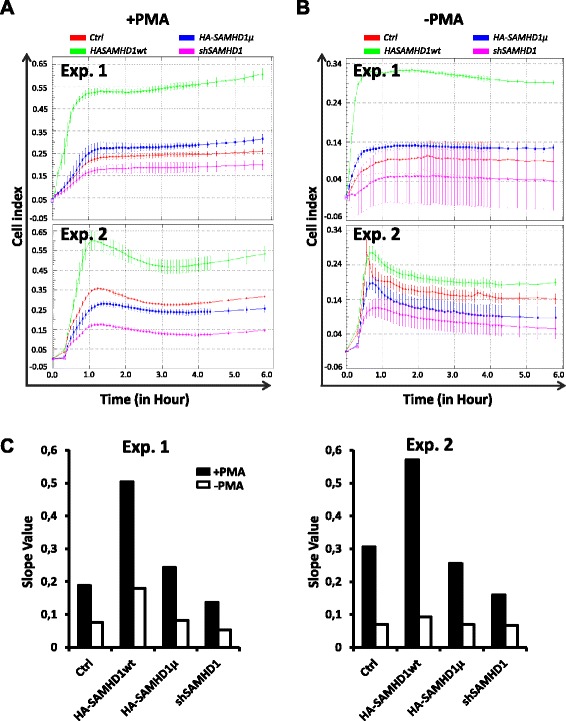
SAMHD1 overexpression increases differentiation-associated adhesion of cells. Equal numbers of cells from each Cell line described in Fig. 1 were tested for their ability to adhere in the presence (**a**) or absence (**b**) of the PMA differentiation-inducer, using the xCELLigence apparatus. Three independant experiments were conducted, two of them are shown. (**c**) The slopes of the curves during the adhesion process in (**a**) and (**b**) (from 0 to 1 h) were calculated for all cell lines in both experiments

Cell adhesion was monitored using the xCELLigence system developed by ACEA Biosciences, which relies on the measurement of electrical impedance of the cell population by micro-electronic biosensors in a plastic well over time [20]. Two parameters reflect fast and strong adhesion: a high slope of the line < impedance = a (time) + b > at the beginning of the measure and a high cell index providing that cells are plated at the same density. Cells overexpressing wt SAMHD1 adhered to the support after PMA addition faster than control cells, SAMHD1 HD/AA expressing cells and SAMHD1 depleted cells whose impedance levels were slightly decreased (Fig. 2a, 2 distinct experiments shown). Same results were obtained with three different clones for each cell line (data not shown and Additional file 1:Figure S1 B for the analysis of the selected clones by western blot). In agreement with these observations, the slope of the line during the first hour of adhesion was higher in cells where SAMHD1 was overexpressed (Fig. 2c). Importantly, SAMHD1 HD/AA overexpression had no effect on the increase of cell adhesion, though it was expressed as well as the wild type protein (see western blot Additional file 1: Figure S1). Finally SAMHD1-depleted cells adhered in a reproducible way a little bit less efficiently than control cells, suggesting that endogenous SAMHD1 could also facilitate differentiation (Fig. 2a).

To our surprise, even in the absence of PMA, a small increase in adhesion capability was detected in SAMHD1 overexpressing cells (Fig. 2b and c). This increase was reproducible but in some experiments was transient (Fig. 2b, Exp. 2) and, in any case, less efficient than in PMA-treated cells (Fig. 2c). Nonetheless, overexpression of wt SAMHD1 was not sufficient to induce cell differentiation or to confer a differentiation-associated restriction phenotype against HIV to dividing THP-1 cells (without PMA), while it can increase restriction when the cells were differentiated in the presence of PMA (data not shown and [21]).

**Fig. 3:**
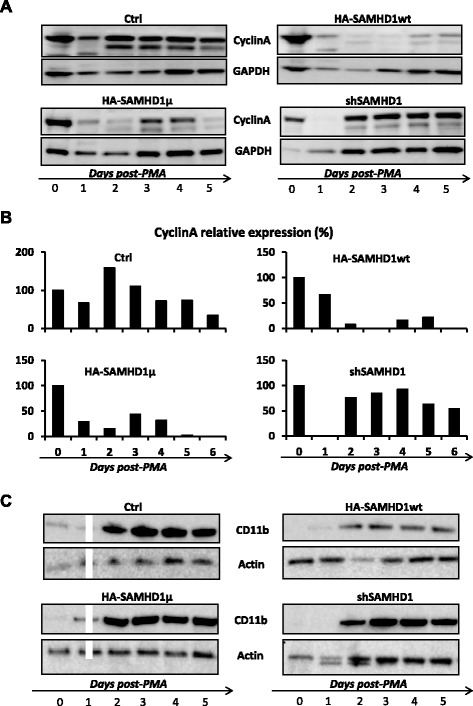
SAMHD1 overexpression contributes to maintain low levels of cyclin A following PMA-induced differentiation. Cell lines described in Fig. 1 received (or not) a 65 nM PMA treatment for 24 h, then were distributed in 6-well plates. Cells were lysed at the indicated times (from day 0 to day 5 of differentiation). Western-blot analysis of two markers was conducted in whole cell extracts: Cyclin A (**a**), whose quantification levels compared to GAPDH are shown in (**b**), and the macrophage differentiation marker CD11b (**c**). The results are representative of two independent experiments

We further analyzed expression of cell cycle (Cyclin A) and differentiation (CD11b) markers following PMA treatment. Cyclin A is a classical cyclin, whose levels fluctuate during cell cycle progression, being up in S and G2 phases and down from early mitosis to the end of G1 and in non-dividing cells [22]. Two to four days after PMA treatment, cyclin A levels remained high in wt THP-1 cells and SAMHD1-depleted cells, but stayed low in SAMHD1 over-expressing cells (Fig. 3a, b and Additional file 3: Figure S3 for a second experiment). No final conclusion could be drawn with SAMHD1 HD/AA cells, in which cyclin A levels did not always fluctuate in the same direction (Fig. 3a, b and Additional file 3: Figure S3). These results further suggest that SAMHD1 could facilitate the quiescent state, though it is unclear whether it depends on the enzymatic activity of the protein. CD11b was also analyzed as a marker of differentiation [15, 16]. The kinetics of CD11b appearance did not significantly change with SAMHD1 over-expression or extinction (Fig. 3c).

**Fig. 4:**
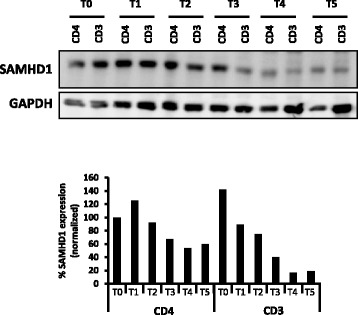
Reduction of SAMHD1 expression after T cell activation. Total peripheral blood CD3+ or CD4+ T cells (extracted from peripheral blood mononuclear cells with BD Bioscience CD3+ or CD4+ negative-selection kit, respectively) were activated by incubation with CD3/CD28 beads (1bead/5cells). Total SAMHD1 levels were assessed in whole cell extracts by western-blot at 0 (T0), 4 (T1) 8 (T2), 24 (T3), 36 (T4), 48 (T5), 72 (T6) hours after activation (top) and normalized to GAPDH for quantification (bottom). One representative experiment among three is shown

Quiescence and differentiation are intimately linked when treating THP-1 cells with PMA. On the one hand entry into quiescence is induced by cellular differentiation. On the other hand, SAMHD1 overexpression may help to maintain a quiescent state that facilitates differentiation. Further work is needed to unravel the exact mechanism by which SAMHD1 impacts this transition process. A distinct relevant model to study transition from quiescence to proliferation *per se* is provided by primary CD4+ T cells, which transit from a quiescent state to a dividing state following activation. This activation process correlates with enhanced cell permissivity to HIV-1 infection and with SAMHD1 phosphorylation [6, 7, 17, 23–25]. We hypothesized that SAMHD1 may contribute to maintain CD4+ T cells in a quiescent state. In support of this hypothesis, we found that SAMHD1 expression is reduced along T-cell activation (Fig. 4) in agreement with recent studies [23]. Future work should aim to analyze whether SAMHD1 overexpression in quiescent CD4+ T cells would delay entry into cell cycle following T cell activation and whether the lentiviral accessory protein Vpx, on the contrary, by triggering SAMHD1 degradation, could accelerate the activation process. We speculate that SAMHD1-mediated restriction finds support in its ability to modify cell cycle parameters, in addition to the direct inhibition of reverse transcription.

## Additional files

Additional file 1: Figure S1. Expression of endogenous and exogenous SAMHD1 in the different cell lines. THP-1 cells transduced with lentiviral vectors expressing HA-tagged SAMHD1 wt, SAMHD1 HD/AA mutant, or shRNA targeting SAMHD1 mRNA were clonally selected under puromycin treatment (2 μg/ml) for two weeks. (A) Expression of HA-SAMHD1 or endogenous SAMHD1 is shown for clones 25 (HA-SAMHD1 wt), 14 (HA-SAMHD1 HD/AA) and 4 (shSAMHD1) that were chosen for the results presented in this manuscript. (B) The results of all the experiments were reproducible with distinct monoclonal cell lines (blue boxes). (PDF 466 kb) Additional file 2: Figure S2. Kinetics of cell morphological changes after PMA addition in the different THP-1 cell lines. Cell lines shown in Fig. 1, differentiated by PMA treatment for 24 h, were observed with a Zeiss 5 microscope (Gx20). Pictures were taken at the indicated times after treatment with PMA. (PDF 610 kb) Additional file 3: Figure S3. Cyclin A level fluctuations after PMA treatment in the different THP-1 cell lines. This figure shows a second independent experiment as the one presented in Fig. 3 a and b. Western-blot assessment of cyclin A and GADPH levels at the indicated times was conducted (A) and quantification was performed (B). (PDF 280 kb)

## References

[1] Hrecka K, Hao C, Gierszewska M, Swanson SK, Kesik-Brodacka M, Srivastava S (2011). Vpx relieves inhibition of HIV-1 infection of macrophages mediated by the SAMHD1 protein. Nature.

[2] Laguette N, Sobhian B, Casartelli N, Ringeard M, Chable-Bessia C, Segeral E (2011). SAMHD1 is the dendritic- and myeloid-cell-specific HIV-1 restriction factor counteracted by Vpx. Nature.

[3] Goldstone DC, Ennis-Adeniran V, Hedden JJ, Groom HC, Rice GI, Christodoulou E (2011). HIV-1 restriction factor SAMHD1 is a deoxynucleoside triphosphate triphosphohydrolase. Nature.

[4] Lahouassa H, Daddacha W, Hofmann H, Ayinde D, Logue EC, Dragin L (2012). SAMHD1 restricts the replication of human immunodeficiency virus type 1 by depleting the intracellular pool of deoxynucleoside triphosphates. Nat Immunol.

[5] Powell RD, Holland PJ, Hollis T, Perrino FW (2011). Aicardi-Goutieres syndrome gene and HIV-1 restriction factor SAMHD1 is a dGTP-regulated deoxynucleotide triphosphohydrolase. J Biol Chem.

[6] Baldauf HM, Pan X, Erikson E, Schmidt S, Daddacha W, Burggraf M (2012). SAMHD1 restricts HIV-1 infection in resting CD4(+) T cells. Nat Med.

[7] Descours B, Cribier A, Chable-Bessia C, Ayinde D, Rice G, Crow Y (2012). SAMHD1 restricts HIV-1 reverse transcription in quiescent CD4+ T-cells. Retrovirology.

[8] Ryoo J, Choi J, Oh C, Kim S, Seo M, Kim SY (2014). The ribonuclease activity of SAMHD1 is required for HIV-1 restriction. Nat Med.

[9] Clifford R, Louis T, Robbe P, Ackroyd S, Burns A, Timbs AT (2014). SAMHD1 is mutated recurrently in chronic lymphocytic leukemia and is involved in response to DNA damage. Blood.

[10] Landau DA, Carter SL, Stojanov P, McKenna A, Stevenson K, Lawrence MS (2013). Evolution and impact of subclonal mutations in chronic lymphocytic leukemia. Cell.

[11] Franzolin E, Pontarin G, Rampazzo C, Miazzi C, Ferraro P, Palumbo E (2013). The deoxynucleotide triphosphohydrolase SAMHD1 is a major regulator of DNA precursor pools in mammalian cells. Proc Natl Acad Sci U S A.

[12] Aird KM, Zhang G, Li H, Tu Z, Bitler BG, Garipov A (2013). Suppression of nucleotide metabolism underlies the establishment and maintenance of oncogene-induced senescence. Cell Rep.

[13] Bester AC, Roniger M, Oren YS, Im MM, Sarni D, Chaoat M (2011). Nucleotide deficiency promotes genomic instability in early stages of cancer development. Cell.

[14] Kunz BA, Kohalmi SE, Kunkel TA, Mathews CK, McIntosh EM, Reidy JA (1994). International commission for protection against environmental mutagens and carcinogens. Deoxyribonucleoside triphosphate levels: a critical factor in the maintenance of genetic stability. Mutat Res.

[15] Munoz-Pacheco P, Ortega-Hernandez A, Miana M, Cachofeiro V, Fernandez-Cruz A, Gomez-Garre D (2012). Ezetimibe inhibits PMA-induced monocyte/macrophage differentiation by altering microRNA expression: a novel anti-atherosclerotic mechanism. Pharmacol Res.

[16] Most J, Schwaeble W, Drach J, Sommerauer A, Dierich MP (1992). Regulation of the expression of ICAM-1 on human monocytes and monocytic tumor cell lines. J Immunol.

[17] Cribier A, Descours B, Valadao AL, Laguette N, Benkirane M (2013). Phosphorylation of SAMHD1 by cyclin A2/CDK1 regulates its restriction activity toward HIV-1. Cell Rep.

[18] Welbourn S, Dutta SM, Semmes OJ, Strebel K (2013). Restriction of virus infection but not catalytic dNTPase activity is regulated by phosphorylation of SAMHD1. J Virol.

[19] White TE, Brandariz-Nunez A, Valle-Casuso JC, Amie S, Nguyen LA, Kim B (2013). The retroviral restriction ability of SAMHD1, but not its deoxynucleotide triphosphohydrolase activity, is regulated by phosphorylation. Cell Host Microbe.

[20] Tibaldi L, Leyman S, Nicolas A, Notebaert S, Dewulf M, Ngo TH (2013). New blocking antibodies impede adhesion, migration and survival of ovarian cancer cells, highlighting MFGE8 as a potential therapeutic target of human ovarian carcinoma. PLoS One.

[21] Dragin L, Nguyen LA, Lahouassa H, Sourisce A, Kim B, Ramirez BC (2013). Interferon block to HIV-1 transduction in macrophages despite SAMHD1 degradation and high deoxynucleoside triphosphates supply. Retrovirology.

[22] Di Fiore B, Davey NE, Hagting A, Izawa D, Mansfeld J, Gibson TJ (2015). The ABBA motif binds APC/C activators and is shared by APC/C substrates and regulators. Dev Cell.

[23] Ruffin N, Brezar V, Ayinde D, Lefebvre C, Schulze Zur Wiesch J, van Lunzen J (2015). Low SAMHD1 expression following T-cell activation and proliferation renders CD4+ T cells susceptible to HIV-1. AIDS.

[24] Schmidt S, Schenkova K, Adam T, Erikson E, Lehmann Koch J, Sertel S (2015). SAMHD1’s protein expression profile in humans. J Leukoc Biol.

[25] Pauls E, Ruiz A, Badia R, Permanyer M, Gubern A, Riveira-Munoz E (2014). Cell cycle control and HIV-1 susceptibility are linked by CDK6-dependent CDK2 phosphorylation of SAMHD1 in myeloid and lymphoid cells. J Immunol.

