# Supplementary figures and images for: Evidence that HIV-1 restriction factor SAMHD1 facilitates differentiation of myeloid THP-1 cells
Source: Virol J. 2015 Nov 25;12:201. doi: 10.1186/s12985-015-0425-y (PMC4660839; doi:10.1186/s12985-015-0425-y)

**A**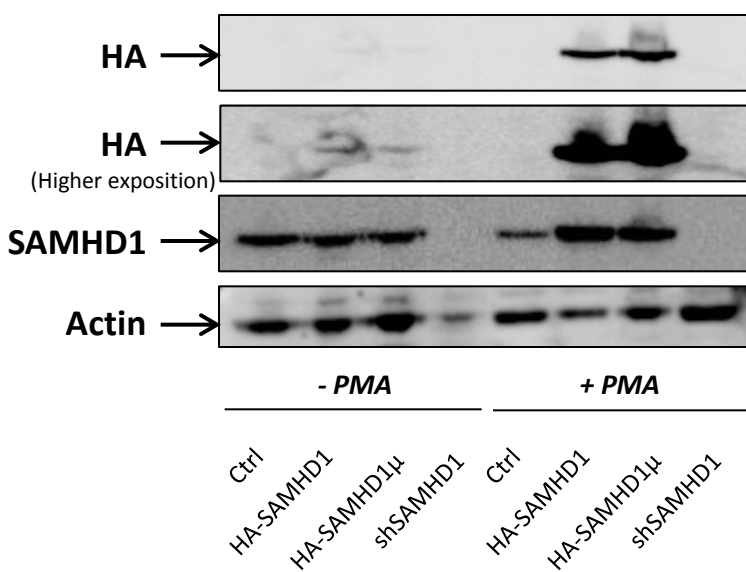**B**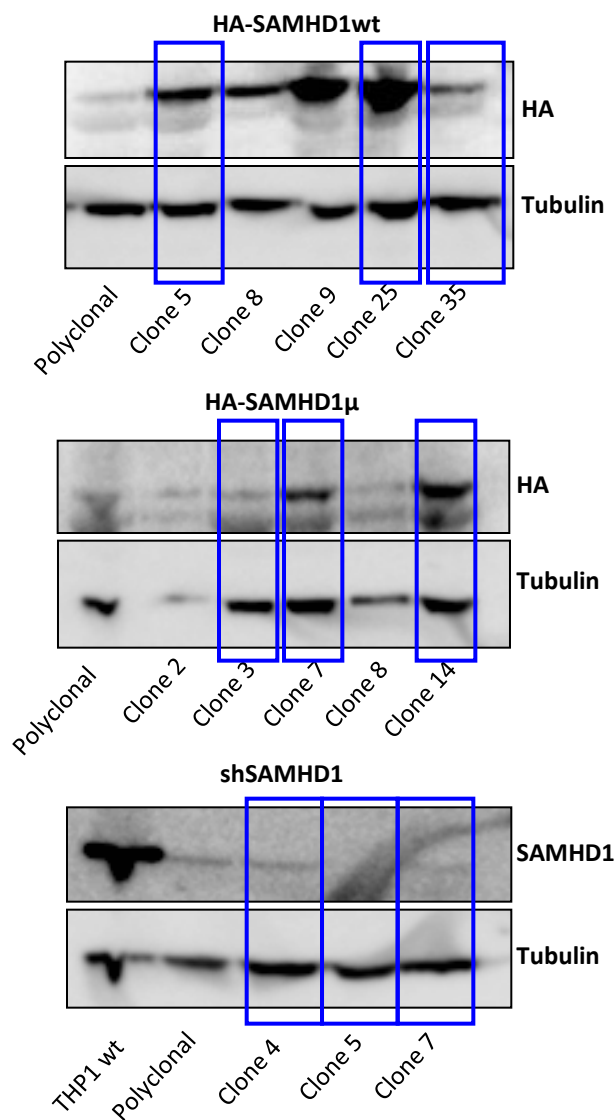**Supplementary Figure 1**

Supplement: Additional file 1: Figure S1. — Expression of endogenous and exogenous SAMHD1 in the different cell lines. THP-1 cells transduced with lentiviral vectors expressing HA-tagged SAMHD1 wt, SAMHD1 HD/AA mutant, or shRNA targeting SAMHD1 mRNA were clonally selected under puromycin treatment (2 μg/ml) for two weeks. (A) Expression of HA-SAMHD1 or endogenous SAMHD1 is shown for clones 25 (HA-SAMHD1 wt), 14 (HA-SAMHD1 HD/AA) and 4 (shSAMHD1) that were chosen for the results presented in this manuscript. (B) The results of all the experiments were reproducible with distinct monoclonal cell lines (blue boxes). (PDF 466 kb) [file 12985_2015_425_MOESM1_ESM.pdf]

**1 day post-PMA**

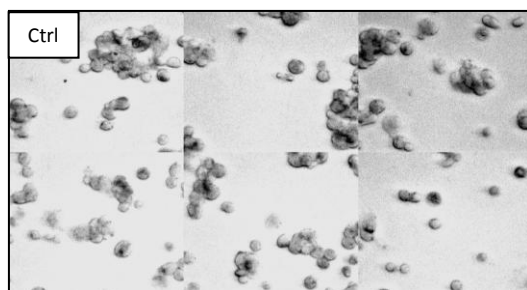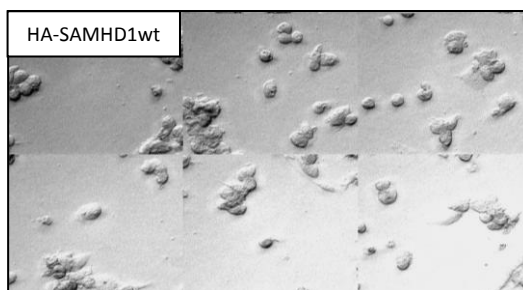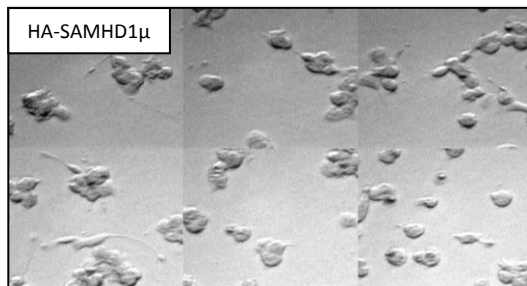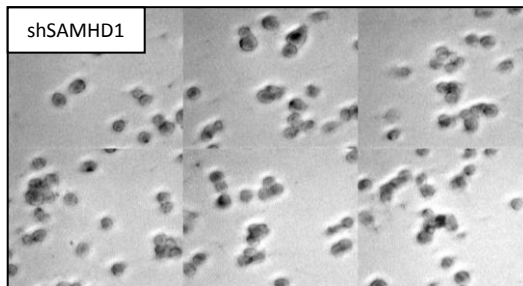

**2 days post-PMA**

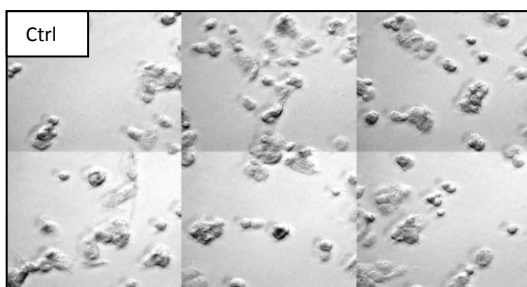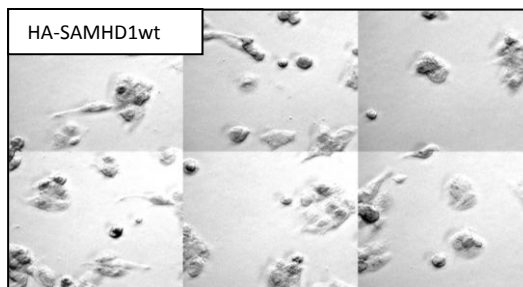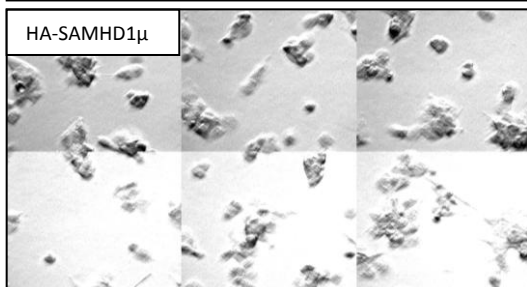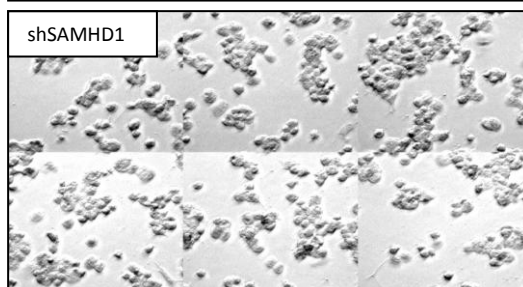

**3 days post-PMA**

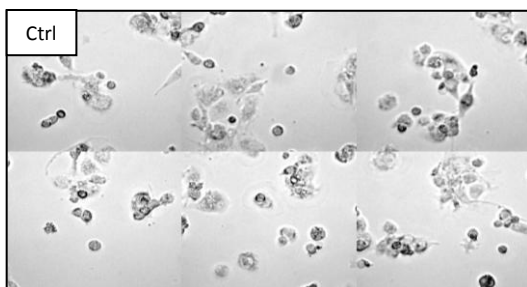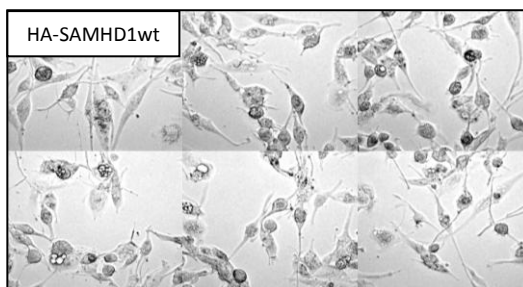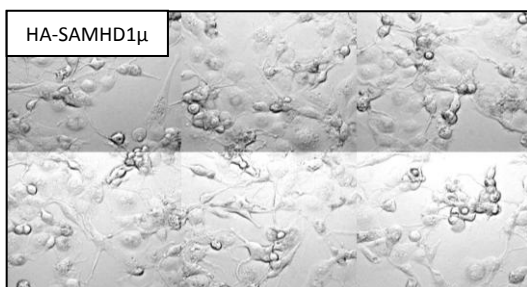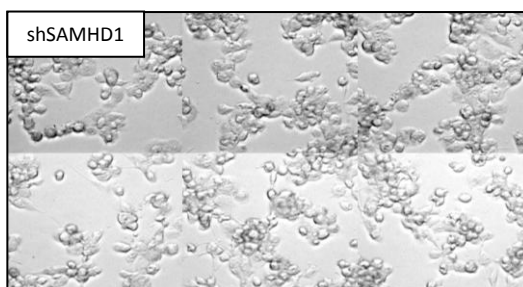

**Supplementary Figure 2**

Supplement: Additional file 2: Figure S2. — Kinetics of cell morphological changes after PMA addition in the different THP-1 cell lines. Cell lines shown in Fig. 1, differentiated by PMA treatment for 24 h, were observed with a Zeiss 5 microscope (Gx20). Pictures were taken at the indicated times after treatment with PMA. (PDF 610 kb) [file 12985_2015_425_MOESM2_ESM.pdf]

A

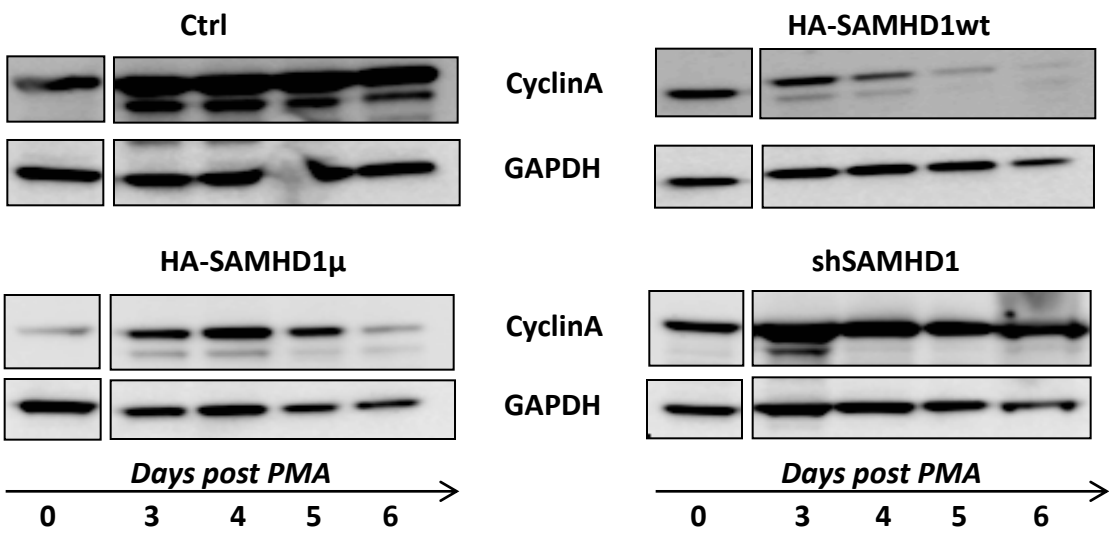

B

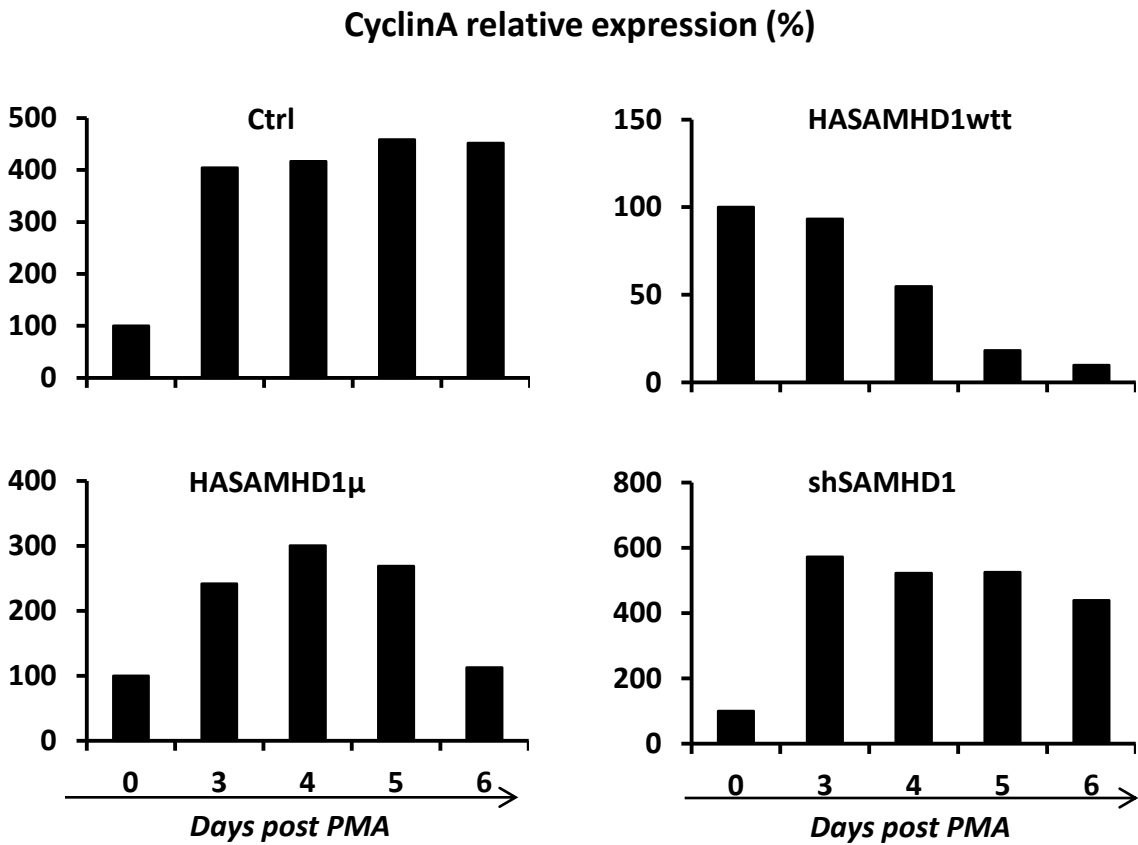

Supplementary Figure 3

Supplement: Additional file 3: Figure S3. — Cyclin A level fluctuations after PMA treatment in the different THP-1 cell lines. This figure shows a second independent experiment as the one presented in Fig. 3 a and b. Western-blot assessment of cyclin A and GADPH levels at the indicated times was conducted (A) and quantification was performed (B). (PDF 280 kb) [file 12985_2015_425_MOESM3_ESM.pdf]
